# Identification, Characterization, and Functional Analysis of Tube and Pelle Homologs in the Mud Crab *Scylla paramamosain*

**DOI:** 10.1371/journal.pone.0076728

**Published:** 2013-10-07

**Authors:** Xin-Cang Li, Xiao-Wen Zhang, Jun-Fang Zhou, Hong-Yu Ma, Zhi-Dong Liu, Lei Zhu, Xiao-Juan Yao, Lin-Gui Li, Wen-Hong Fang

**Affiliations:** 1 Key Laboratory of East China Sea and Oceanic Fishery Resources Exploitation and Utilization, Ministry of Agriculture, Shanghai, P. R. China; 2 East China Sea Fisheries Research Institute, Chinese Academy of Fishery Sciences, Shanghai, P. R. China; 3 Scholl of Life Science, Henan Normal University, Xinxiang, P. R. China; Uppsala University Evolutionary Biology Center, Sweden

## Abstract

Tube and Pelle are essential components in *Drosophila* Toll signaling pathway. In this study, we characterized a pair of crustacean homologs of Tube and Pelle in *Scylla paramamosain*, namely, *Sp*Tube and *Sp*Pelle, and analyzed their immune functions. The full-length cDNA of *SpTube* had 2052 bp with a 1578 bp open reading frame (ORF) encoding a protein with 525 aa. A death domain (DD) and a kinase domain were predicted in the deduced protein. The full-length cDNA of *SpPelle* had 3825 bp with a 3420 bp ORF encoding a protein with 1140 aa. The protein contained a DD and a kinase domain. Two conserved repeat motifs previously called Tube repeat motifs present only in insect Tube or Tube-like sequences were found between these two domains. Alignments and structure predictions demonstrated that *Sp*TubeDD and *Sp*PelleDD significantly differed in sequence and 3D structure. Similar to TubeDD, *Sp*TubeDD contained three common conserved residues (R, K, and R) on one surface that may mediate *Sp*MyD88 binding and two common residues (A and A) on the other surface that may contribute to Pelle binding. By contrast, *Sp*PelleDD lacked similar conservative residues. *Sp*Tube, insect Tube-like kinases, and human IRAK4 were found to be RD kinases with an RD dipeptide in the kinase domain. *Sp*Pelle, Pelle, insect Pelle-like kinases, and human IRAK1 were found to be non-RD kinases lacking an RD dipeptide. Both *SpTube and SpPelle* were highly expressed in hemocytes, gills, and hepatopancreas. Upon challenge, *SpTube* and *SpPele* were significantly increased in hemocytes by Gram-negative or Gram-positive bacteria, whereas only *SpPelle* was elevated by *White Spot Syndrome Virus*. The pull-down assay showed that *Sp*Tube can bind to both *Sp*MyD88 and *Sp*Pelle. These results suggest that *Sp*Tube, *Sp*Pelle, and *Sp*MyD88 may form a trimeric complex involved in the immunity of mud crabs against both Gram-negative and Gram-positive bacteria.

## Introduction

Toll and/or Toll-like receptors (TLRs) are membrane protein molecules evolutionarily conserved from insects to mammalian [1]. As crucial pattern recognition receptors, TLRs can be activated directly by recognizing distinct pathogen-associated molecular patterns (PAMPs) or indirectly by a cascade reaction triggered by pathogens [2]. In *Drosophila*, the Toll signaling pathway serves a major function in the defense against Gram-positive bacteria and fungi because it can be activated indirectly by interacting with the proteolytically cleaved ligand Spaetzle [3], [4]. Moreover, the diaminopimelic acid-type peptidoglycan of Gram-negative bacteria can also activate the Toll signaling pathway and further generate antibacterial peptides [5]. Aside from Toll, only Toll7 out of the eight other *Drosophila* TLRs has been recently proven to be involved in immunity, participating in antiviral autophagy in *Drosophila* [6]. By contrast, most mammalian TLRs are involved in different immune signaling pathways through sensing distinct PAMPs. TLR5, TLR11, TLR4, and the heterodimers of TLR2–TLR1 or TLR2–TLR6 located at the cell surface can be activated by recognizing bacterial flagellin, uropathogenic bacteria, bacterial LPS, and bacterial lipoproteins, respectively. Meanwhile, TLR3, TLR7–TLR8, TLR9, and TLR13 reside at the surface of endosomes, where they can be trigged by sensing double-stranded viral RNA, single-stranded viral RNA, CpG-rich bacterial or viral DNA, and bacterial ribosomal RNA, respectively [7].

The adaptor Tube (*Drosophila melanogaster* Tube or DmTube) and kinase Pelle (*D. melanogaster* Pelle or DmPelle) are essential components of the Toll signaling pathway in *Drosophila*. Upon ligand binding, dMyD88 (*D. melanogaster* MyD88), an inflexible regulator anchoring at PIP2-rich regions of the plasma membrane, can recruit the activated Toll receptor and the cytosolic adaptor Tube to permit Toll signaling [8], [9]. Subsequently, Pelle is recruited to the vicinity of the Tube, thereby forming a trimeric complex (dMyD88-Tube-Pelle) mediated by the Tube death domain (DD) [10]. This process activates Pelle by autophosphorylation and its disassociation from this complex [11]. Similarly, IRAK4 and IRAK1, the pair homologs of Tube and Pelle, are essential components of human TLR signaling pathways. IRAK4 can also be recruited by MyD88; hence, these two proteins form a complicated scaffold to recruit IRAK1. Finally, IRAK1 is activated with the involvement of IRAK4 and then released from this complex [12]. However, compared with Tube, IRAK4 with the C-terminal kinase domain is a protein kinase. Thus, it can more elaborately modulate the autophosphorylation activation of IKAK1 by dint of this kinase domain [13].

Studies on the innate immunity of crustaceans have attracted considerable attention because of the huge losses caused by various aquatic animal diseases in the past decades [14], [15]. Currently, increasing evidence implies that TLR signaling pathways consisting of the homologs of TLR/MyD88/Tube/Pelle/TRAF6/NF-κB in *Drosophila*, which are involved in the defense against pathogen invasion, may exist in crustaceans. As the central components of TLR signaling pathways, TLRs have been identified in various crustaceans and have been proven to participate in immunity and defense against several pathogens [16]–[20]. Furthermore, the homologs of most other components in the Toll signaling pathway, such as MyD88, TRAF6, Cactus, and Dorsal (nuclear factor-κB), have also been characterized [21]–[24]. However, two essential components of Tube and Pelle homologs in crustaceans have not been identified or well-characterized. In addition, the mechanism by which these TLR signaling pathways function remains unclear.

In our previous work, we proposed a possible antibacterial model mediated by *Sp*Toll and *Sp*MyD88 in mud crab [25]. In this model, *Sp*MyD88 pre-docking at specific sites of the plasma membrane with its C-terminal extension (CTE) domain may recruit activated *Sp*Toll. Thus, *Sp*MyD88 cooperating with *Sp*Toll is involved in immunity against Gram-negative bacteria. In this study, we further identified the downstream components of *Sp*MyD88, namely, *Sp*Tube and *Sp*Pelle, a pair of homologs of Tube and Pelle in mud crabs, and analyzed their individual characteristics and likely immune functions.

## Materials and Methods

### Immune Challenge and Tissue Collection

Mud crabs (150 g to 180 g each) obtained from a farm in Chongming County (Shanghai, China) were cultured temporarily in flowing, aerated seawater in 400 L tanks in laboratory (This study did not involve in endangered or protected species, and no specific permissions were required for the animal experiments). The expression profiles after injecting 50 µL of *Vibrio harveyi* (2×10^7^ CFU), 50 µL of *Staphylococcus aureus* (2×10^8^ CFU) suspension, or 100 µL of *White Spot Syndrome Virus* (WSSV) inoculum (1×10^5^ copies) into the base of the right fifth leg of each crab were investigated. The control was challenged with 50 µL of phosphate-buffered saline (140 mM NaCl and 10 mM sodium phosphate; pH 7.4) or with the supernatant of normal tissues. After anesthetizing the crabs with ice for 15 min, hemolymph was collected from the base of the right chelate leg with an ice-cold anticoagulant buffer (0.14 M NaCl, 0.1 M glucose, 30 mM trisodium citrate, 26 mM citric acid, and 10 mM ethylenediaminetetraacetic acid; pH 4.6) [26] at 0, 2, 6, 12, 24, and 48 h after challenge with *V. harveyi*, *S. aureus*, or WSSV. The collected samples were centrifuged immediately at 800×*g* for 15 min at 4°C to isolate the hemocytes. The gill, hepatopancreas, heart, stomach, intestine, connective tissue, muscle, and eyestalk of the mud crabs were also collected for RNA isolation. At least three crabs from each sample were selected to eliminate individual differences.

### RNA Extraction and cDNA Synthesis

The collected tissues from normal crabs, together with the hemocytes from normal or pathogen-challenged crabs at different times, were utilized to isolate the total RNA with TRIzol Reagent (Ambion, USA) following the manufacturer’s instructions. First-strand cDNA was synthesized with 5 µg of RNA as the template using a First-strand cDNA Synthesis kit according to the manufacturer’s instructions.

### cDNA Cloning

A cDNA fragment of *SpTube* and a partial *SpPelle* cDNA sequence were identified by high-throughput transcriptome sequencing with a mixture of hemocytes and hepatopancreas. Four pairs of primers (*Sp*TubeF1 and *Sp*TubeR1; *Sp*TubeF2 and *Sp*TubeR2; *Sp*PelleF1 and *Sp*PelleR1; *Sp*PelleF2 and *Sp*PelleR2) were designed for polymerase chain reaction (PCR) and for verifying the harvested *SpTube* and *SpPelle* fragments. Two additional primers (*Sp*TubeCF and *Sp*PelleCF) paired with a 3′ anchor R primer were also synthesized to further amplify the cDNA sequence 3′ ends of *SpTube* and *SpPelle*, respectively. PCR was conducted under the following parameters: 94°C for 2 min; 35 cycles of 94°C for 35 s, 53°C for 45 s, and 72°C for 50 s; and 72°C for 10 min. The corresponding amplified fragments were subcloned into pMD-18T vectors prior to sequencing by a commercial company (Sangon, China). Complete cDNA sequences were obtained by overlapping their fragments of individual genes.

### Bioinformatics Analysis

Alignments of amino acid sequences were performed using the ClustalX 2.0 program (http://www.ebi.ac.uk/tools/clustalw2) and GenDoc software. Online Basic Local Alignment Search Tool Program (BLASTP) (http://blast.ncbi.nlm.nih.gov/Blast.cgi) was used to analyze protein similarities. Translation of the amino acid sequence and prediction of the deduced protein were conducted on http://web.expasy.org/translate/. The putative domain was predicted by the Simple Modular Architecture Research Tool (http://smart.embl-heidelberg.de/). Theoretical isoelectric point (pI) and molecular weight (Mw) were calculated with online software (http://web.expasy.org/compute_pi/). MEGA4.0 was used to construct phylogenetic trees, and 1,000 bootstraps were selected for the neighbor-joining tree to assess reliability [27]. The three-dimensional (3D) structure of *Sp*Tube DD was predicted through the Phyre server [28] using the crystal structure of DmTube DD (PDB ID: 1d2z, chain B) as template. The 3D structure of *Sp*Pelle DD was modeled by homology using the SWISS-MODEL workspace (http://swissmodel.expasy.org/workspace/) based on the templates of the solution structure of DmPelle DD (PDB ID: 1d2z, chain A). Using the same SWISS-MODEL online software, the 3D structures of both *Sp*Tube and *Sp*Pelle kinase domains were also predicted based on the crystal structure of the human IRAK4 kinase domain (PDB ID: 2nru, chain B). Comparative modeling of the 3D structures of DD or the kinase domain was generated by Swiss-PdbViewer V 4.0 software [29].

### Real-time PCR

Using the synthesized cDNA as template, quantitative reverse transcription PCR (qRT-PCR) was conducted to examine the relative expression levels of *SpTube* and *SpPelle* in a real-time thermal cycler (ABI, USA) based on a previous protocol [25]. Two pairs of primers (*Sp*TubeRF and *Sp*TubeRR; *Sp*PelleRF and *Sp*PelleRR) were used to generate 191 and 147 bp amplicons, respectively (Table 1). Another pair of primers (18SRF and 18SRR) was used to amplify the corresponding fragment (121 bp) as reference. The total volume was 20 µL (10 µL of 2× Premix Ex Taq, 2 µL of cDNA, and 4 µL of each primer). qRT-PCR was programmed as follows: 95°C for 5 min; 40 cycles of 95°C for 10 s and 60°C for 50 s; and a melt from 60°C to 95°C. All tests were conducted thrice using individual templates. The relative expression levels of *SpTube* and *SpPelle* in different tissues were calculated according to the 2^−△CT^ method. The algorithm of 2^−△△CT^ was applied in expression profile analysis.

**Table 1:** Sequences of the primers used in this study.

Primer	Sequence (5′-3′)
*Sp*Tube	
*Sp*TubeF1	TTTCCTTACTGTGTATATGT
*Sp*TubeR1	CACCTTCTTCTCTCTACCC
*Sp*TubeF2	CTGGATTTTGAGGTCGTGC
*Sp*TubeR2	CCTTCTCGCTCCTCTTGGTA
*Sp*TubeCF	TGCCACCCTCATCAAGACCA
*Sp*TubeRF	AACTGGATTTTGAGGTCGTGC
*Sp*TubeRR	CACCTTCTTCTCTCTACCCCCTA
*Sp*TubeDDEF	TACTCAGAATTCGTGACACTGACCTCAGAGCT
*Sp*TubeDDER	TACTCACTCGAGTTAAACAGTGACAATGAGATA
*Sp*Pelle	
*Sp*PelleF1	GAGTGAGGGTGTTGGTGCCA
*Sp*PelleR1	AGTGATGTGGAGACGGGTGTTA
*Sp*PelleF2	CCAGTGATTCCCTACAAAGAGC
*Sp*PelleR2	GTTCATGTTGAGGCTTGACAGCT
*Sp*PelleCF	GCAACAGGATCCTCCTAGCTAT
*Sp*PelleRF	ACATCTGGATAACACCCGTCTC
*Sp*PelleRR	GGCATGAACTGGTACACAAGG
*Sp*PelleDDEF	TACTCAGAATTCGTGAAGTACGTGTACGACT
*Sp*PelleDDER	TACTCACTCGAGTTAAACACAAGACTTGAGTGCT
*Sp*MyD88	
*Sp*MyD88DDEF	TACTCAGAATTCTCCACAAGAAAGCACATG
*Sp*MyD88DDER	TACTCACTCGAGTTATATCATATCCCTTGTATCAT
Universal Primer	
3′anchor R	GACCACGCGTATCGATGTCGAC
18sRNA	
18SF	CAGACAAATCGCTCCACCAAC
18SR	GACTCAACACGGGGAACCTCA

Underlined nucleotides indicate the locations of the restricted endonucleases.

### Recombinant Expression and Purification

According to the full-length cDNA sequences of *Sp*MyD88, *Sp*Tube, and *Sp*Toll, three primer pairs (*Sp*MyD88DDEF and *Sp*MyD88DDER; *Sp*TubeDDEF and *Sp*TubeDDER; *Sp*PelleDDEF and *Sp*PelleDDER) were designed to amplify the sequences that encode their corresponding fragments, respectively (Table 1). The amplified fragment of *Sp*MyD88DD or *Sp*PelleDD was digested by *Eco*R I and *Xho* I, and then inserted into a pET−30a (+) vector. Meanwhile, the *Sp*TubeDD fragment was ligated into a pGEX4T1 expression vector after being cut by *Eco*R I and *Xho* I. Three recombinant expression vectors, namely, pET-30a-MyD88DD, pET-30a-PelleDD, and pGEX4T1-TubeDD, were then transformed into competent *Escherichia coli* Rosetta (DE3) host cells. Isopropyl-β-D-thio-galactoside (IPTG) was added to induce protein expression. The recombinant *Sp*TubeDD protein with a glutathione S-transferase (GST) tag and a GST protein was purified using glutathione Sepharose 4B chromatography (GenScript, USA) according to the manufacturer’s instructions. Both *Sp*MyD88DD and *Sp*PelleDD proteins with an N-terminal His tag were harvested by His Bind resin chromatography (Novagen, USA) according to a previous method [30].

### Pull-down Assay

Pull-down assay was performed according to a previous protocol with slight modifications [31]. Approximately 1 mL of purified *Sp*MyD88DD or *Sp*PelleDD (200 µg/mL) was incubated with His-Bind resin (1 mL) for 10 min at 4°C and then washed with 6 mL of binding buffer (0.5 M NaCl, 20 mM Tris-Cl pH 7.9, 5 mM imidazole). Afterward, *Sp*TubeDD (1 mL) was added and then incubated for 30 min at 4°C. After being washed thoroughly with wash buffer (0.5 M NaCl, 20 mM Tris-Cl pH 7.9, 60 mM imidazole), the proteins were eluted with elution buffer (0.5 M NaCl, 20 mM Tris-Cl pH 7.9, 1 M imidazole) and then finally analyzed on 15% sodium dodecyl sulfate-polyacrylamide gel electrophoresis (SDS-PAGE). GST protein was used as the negative control in this assay.

### Statistical Analysis

Significant differences were determined by one-way analysis of variance and Duncan’s test for multiple range comparison using SPSS 13.0, with significant levels accepted at *P*<0.05. All experiments were repeated at least three times. Data are shown as means ± standard deviation.

## Results

### cDNA Cloning

**Figure 1:**
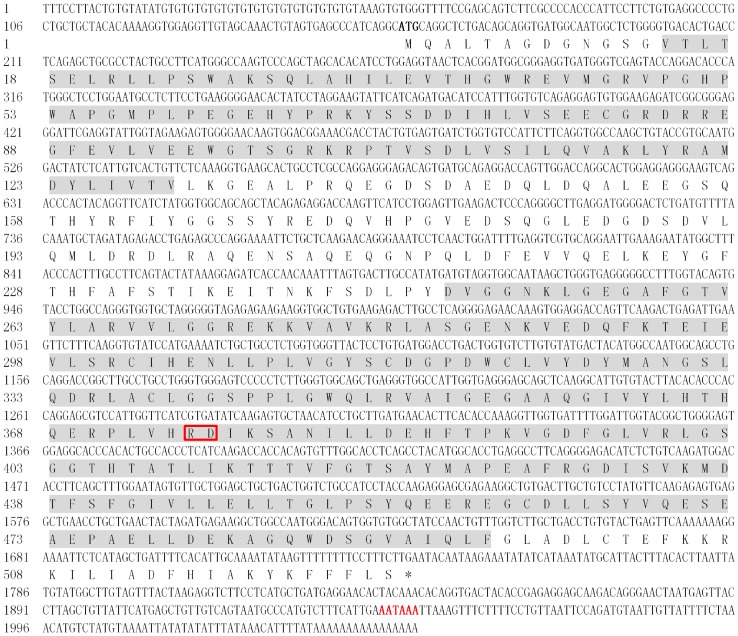
Full-length cDNA and translated amino acid sequences of *Sp*Tube. The death domain (the first domain) and kinase domain (the second domain) are shadowed. The stop codon is indicated by an asterisk (*). The initiation codon and the polyadenylation signal are shown in bold and red letters, respectively. The RD dipeptide existing only in Tube-like kinases but not in Pelle or Pelle-like protein sequences is marked with red box.

**Figure 2:**
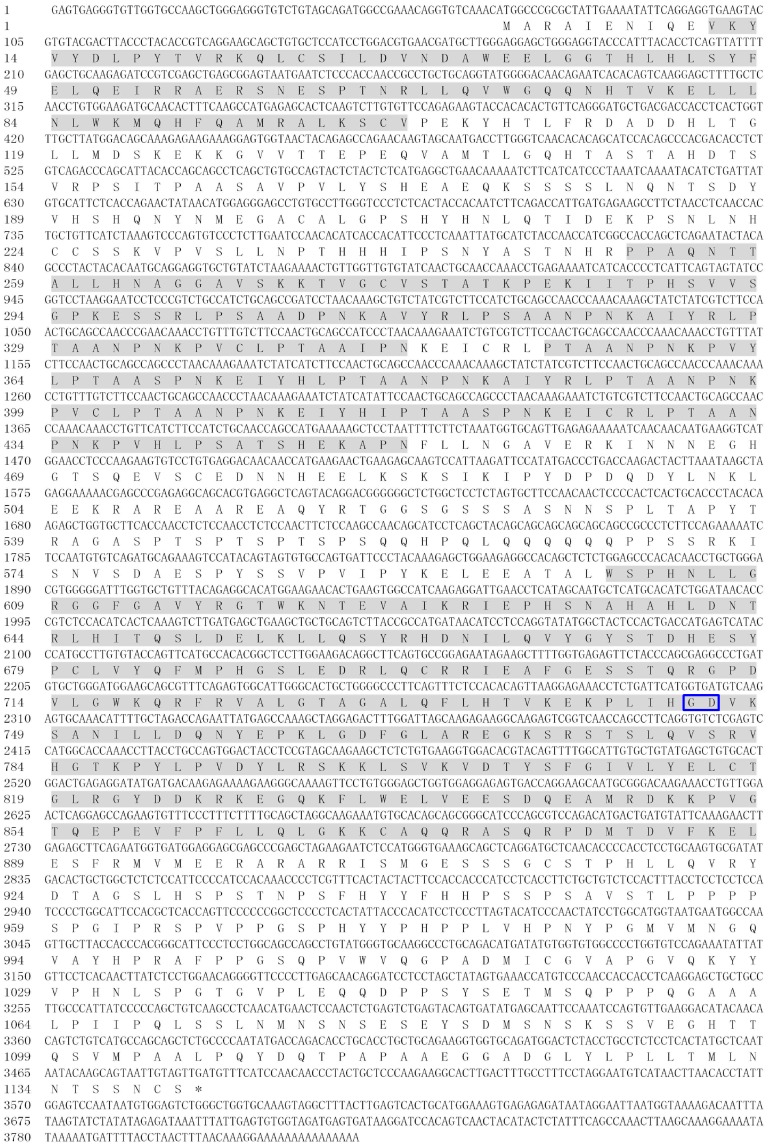
Complete cDNA and translated amino acid sequences of *Sp*Pelle. The death domain (first domain), two conserved repeat motifs (two middle sequences), and kinase domain (last domain) are shadowed orderly. The stop codon is indicated by an asterisk (*). GD dipeptide rather than RD dipeptide is shown in blue frame.

A *SpTube* fragment and a partial *SpPelle* cDNA sequence harvested from transcriptome sequencing were verified further by PCR using the synthesized cDNA as template. Based on these two partial cDNA sequences, the 3′ ends of the complete sequences were obtained through the Rapid Amplification of cDNA Ends technology. The full-length cDNA sequences of *SpTube* and *SpPelle* were determined by overlapping their individual fragments. The complete cDNA sequence of *SpTube* had 2052 bp, with a 159 bp 5′ untranslated region (UTR), a 315 bp 3′ UTR, and a 1578 bp open reading frame (ORF) encoding a protein with 525 aa (Genbank Accession No. KF155697) (Fig. 1). A DD (residues 14 to 129) and a serine/threonine kinase domain (residues 248 to 495) were predicted in the deduced protein; no signal peptide was found. The theoretical pI and Mw of *Sp*Tube were 4.91 and 58.2 kDa, respectively. The complete sequence of *SpPelle* had 3825 bp, with a 3420 bp ORF encoding a protein with 1140 aa, a 65 bp 5′ UTR, and a 337 bp 3′ UTR with a poly(A) tail (Genbank Accession No. KF155698). The nucleotide and deduced amino acid sequences are shown in Fig. 2. A DD (residues 11 to 101), two repeat motifs (residues 252 to 346, and residues 353 to 451), and a serine/threonine kinase domain (residues 601 to 888) were found in the deduced protein sequence. No signal peptide was predicted. The mature *Sp*Pelle peptide had a theoretical Mw of 125.5 kDa and a pI of 8.22.

### Similarity and Phylogenetic Analyses

The BLASTP search analysis demonstrated that *Sp*Tube shared the highest identity (65%) with *Litopenaeus vannamei* Pelle homologs (AEK86521) at the amino acid level. *Sp*Tube also shared 46% identity with *Homo sapiens* IRAK4 (interleukin-1 receptor associated kinase 4, EAW57867), 46% with *Tribolium castaneum* EFA09756, 38% with *Daphnia pulex* EFX85081, and 37% with *Crassostrea gigas* EKC43058. In addition, *Sp*Pelle shared 50% identity with *D. pulex* Pelle EFX66709, 46% with *T. castaneum* Pelle EFA05720, 43% with *D. melanogaster* Pelle AAF56686, and 42% with *H. sapiens* IRAK1 AAH14963. Although these two proteins exhibited similar structure elements, *Sp*Tube only shared 12.37% identity with *Sp*Pelle.

**Figure 3:**
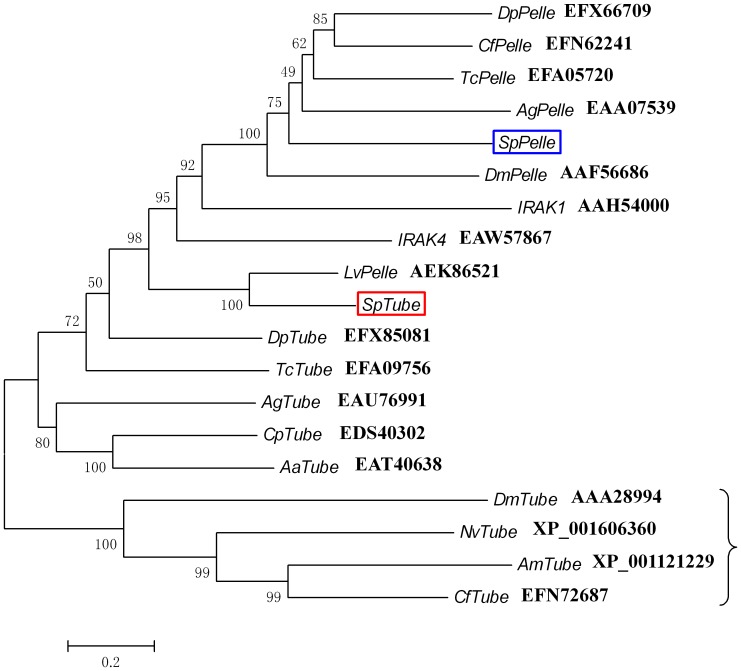
Phylogenetic analysis of the retrieved Tube and Pelle homologs from insects, crustaceans, and humans. NJ trees were generated using the MEGA 4.0 software. The corresponding GenBank accession numbers are listed in this figure. *Sp*Tube and *Sp*Pelle are marked with red and blue frames, respectively. *Dm, Drosophila melanogaster; Cp, Culex pipiens; Aa, Aedes aegypti; Ag, Anopheles gambiae; Nv, Nasonia vitripennis; Dp, Daphnia pulex; Am, Apis mellifera; Cf, Camponotus floridanus; Tc, Tribolium castaneum; Lv, Litopenaeus vannamei; Hs, Homo sapiens; Sp, Scylla paramamosain*.

Based on the BLASTP results, a phylogenetic tree was constructed with Tube and Pelle homologs from insects, crustaceans, and humans to identify the evolutionary relationship among these homologs (Fig. 3). In this tree, Tube homologs without Kinase domain from *D. melanogaster*, *Camponotus floridanus*, *Apis Mellifera*, and *Nasonia vitripennis* formed a unique group, whereas other Tube and Pelle homologs were grouped into a large cluster. In this large group, all Pelle homologs (except for *Lv*Pelle) and IRAK1 formed a small meaningful cluster (the value at the nodes of the subgroups is over 70), suggesting that these Pelle homologs, including *Sp*Pelle, showed closer evolutionary relationship with human IRAK1. In addition, the *Sp*Tube and *Lv*Pelle in this tree formed another small cluster, and these two protein molecules exhibited closer evolutionary relationship with human IRAK4.

### Alignments of DD and Kinase Domains

**Figure 4:**
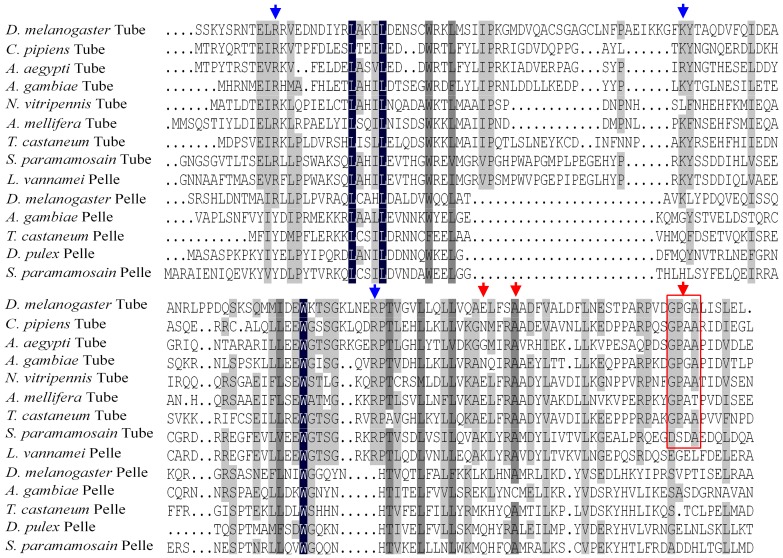
Alignment of the death domains of the Tube and Pelle homologs from insects and crustaceans. The death domains of *Sp*Tube and *Drosophila* Tube share three conserved core residues (R, K, and R, indicated by blue arrows) required for binding to dMyD88. The death domains of Insect Tube homologs contain several common conserved core residues (E, A, GPXA, indicated by red arrows) required for binding to Pelle. The death domains of *Sp*Tube and *Drosophila* Tube share two core residues (A, A).

**Figure 5:**
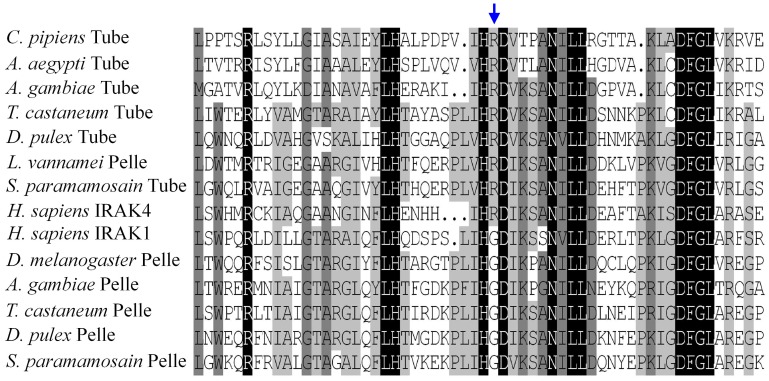
Alignment of sequence subdomains of the Tube and Pelle kinase domains. Arrow indicates the position of RD dipeptide, which is the hallmark of kinases regulated by activation loop phosphorylation. Tube-like kinases, but not Pelle or Pelle-like proteins, are RD kinases.

Alignments of DD sequences demonstrated that Tube and Pelle DD sequences showed clear and significant differences. Similar to Tube DD, the DDs of most insect Tube-like proteins contained three common conserved residues (R, K, and R) required for binding to MyD88s and several residues (E, A, GPXA) essential for binding to Pelles. *Sp*Tube and Tube shared the same three conserved residues (R, K, and R) that mediate binding to dMyD88 and two strongly conserved residues (A and A) that mediate binding to Pelle. By contrast, Pelle and Pelle-like protein sequences, except for *Lv*Pelle, lacked the said conserved residues (Fig. 4). In addition, alignments of kinase domain sequences demonstrated that these proteins can be classified as RD or non-RD kinases based on a single position in sequence, which has been accepted as a criterion for distinguishing these two types of protein kinase [32]. Arthropod Tube-like kinases, human IRAK4, and *Lv*Pelle kinase were RD kinases with an RD dipeptide in the kinase domain; meanwhile, *Sp*Pelle, Pelle, arthropod Pelle-like kinases, and human IRAK1 were non-RD kinases lacking an RD dipeptide (Fig. 5).

### Comparisons of the Predicted 3D Structures

**Figure 6:**
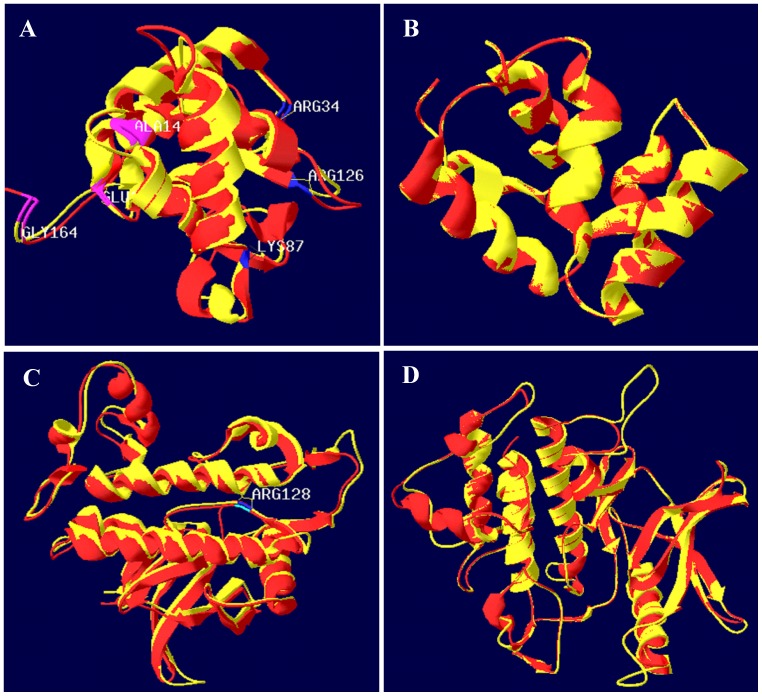
Comparisons of the predicted 3D structure of the *Sp*Tube death domain (DD). (14 aa to 144 aa, yellow) and the crystal structure of DmTube DD (PDB ID: 1d2z, chain B) (red) (A); the predicted 3D structure of *Sp*Pelle DD (13 aa to 109 aa, yellow) and the solution structure of DmPelle DD (PDB ID: 1d2z, chain A) (red) (B); the predicted 3D structure of *Sp*Tube kinase domain (251 aa to 488 aa, yellow) and the crystal structure of human IRAK4 kinase domain (PDB ID: 2nru, chain B) (red) (C); and the predicted 3D structure of *Sp*Pelle kinase domain (540 aa to 825 aa, yellow) and the crystal structure of human IRAK4 kinase domain (PDB ID: 2nru, chain B) (red) (D). Residues required for binding to MyD88 (blue) and Pelle (red) are indicated (A). The position of R (ARG) residue in RD dipeptide, which is the hallmark of Tube-like kinases, is also shown (C). These four predicted structures merge well with their corresponding templates.

Pairwise sequence alignments showed that the DDs of *Sp*Tube and *Sp*Pelle shared 28% and 35% identities with Tube and Pelle DDs, respectively. The *Sp*Tube kinase domain shared 43% identity with the human IRAK4 kinase domain, whereas the *Sp*Pelle kinase domain shared only 34% identity with its IRAK4 counterpart. Thus, the SWISS-MODEL workspace was utilized to construct the 3D models of the kinase domains of *Sp*Tube and *Sp*Pelle, as well as the DD of *Sp*Pelle, because of the high identities with their corresponding individual templates (more than 30%). Given that the identity between the two DDs of *Sp*Tube and DmTube was below 30%, Phyre server was recommended for predicting the 3D model. The *Sp*Tube DD (from 14 aa to 144 aa) and *Sp*Pelle DD (from 13 aa to 109 aa) aligned well with the crystal structure of Tube and Pelle DDs, respectively (Figs. 6A and 6B). Furthermore, similar to the spatial location of the three conserved core residues interacting with dMyD88 at one binding surface and that of several core residues interacting with Pelle at the other in Tube DD, *Sp*Tube DD also had several common conserved core residues at two separate binding surfaces with similar spatial locations. In addition, both the *Sp*Tube kinase domain (from 251 aa to 488 aa) and *Sp*Pelle kinase domain (from 540 aa to 825 aa) can merge well with the crystal structure of the human IRAK4 kinase domain (Figs. 6C and 6D). These findings suggest that these DDs and kinase domains may have similar structures and further exhibit similar functions with their corresponding counterparts in fruitfly or human.

### Structure Elements in Tube and Pelle Homologs

**Figure 7:**
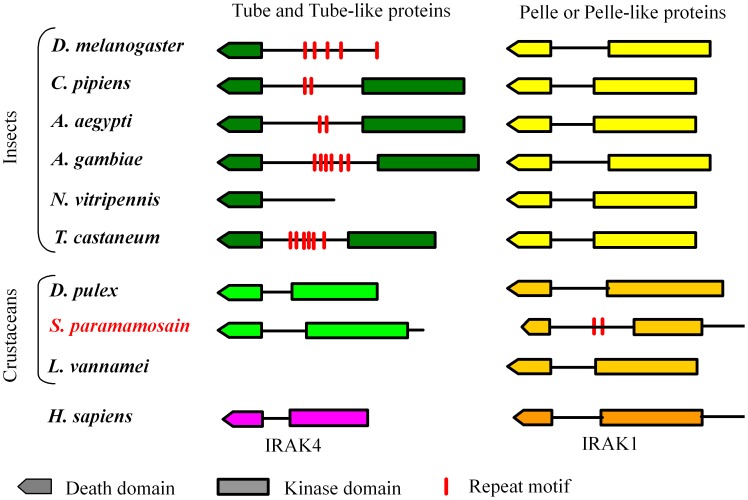
Diagram of the structural elements of Tube and Pelle homologs. *D. melanogaster* Tube and *N. vitripennis* Tube-like proteins lack their individual kinase domains. Repeat motifs are observed in both insect Tube and Tube-like proteins and *Sp*Pelle.

Based on the diagrams of Tube and Pelle homologs (Fig. 7), most of the Tube and Pelle homologs, including human IRAK4 and IRAK1, were identified as kinase proteins containing a DD and a kinase domain. *D. melanogaster* and *N. vitripennis* Tube sequences were non-kinase proteins lacking a kinase domain. In addition, the “Tube repeat motif” present in most insect Tube homologs but not in insect Pelle homologs was absent in crustacean Tube homologs (*Sp*Tube and *Dp*Tube) and human IRAK4. However, this motif was present in the *Sp*Pelle sequence. These two motifs that share 43% identity with each other were the first identified “Tube repeat motifs” in Pelle homologs.

### Tissue Distributions and Expression Profiles After Challenge

**Figure 8:**
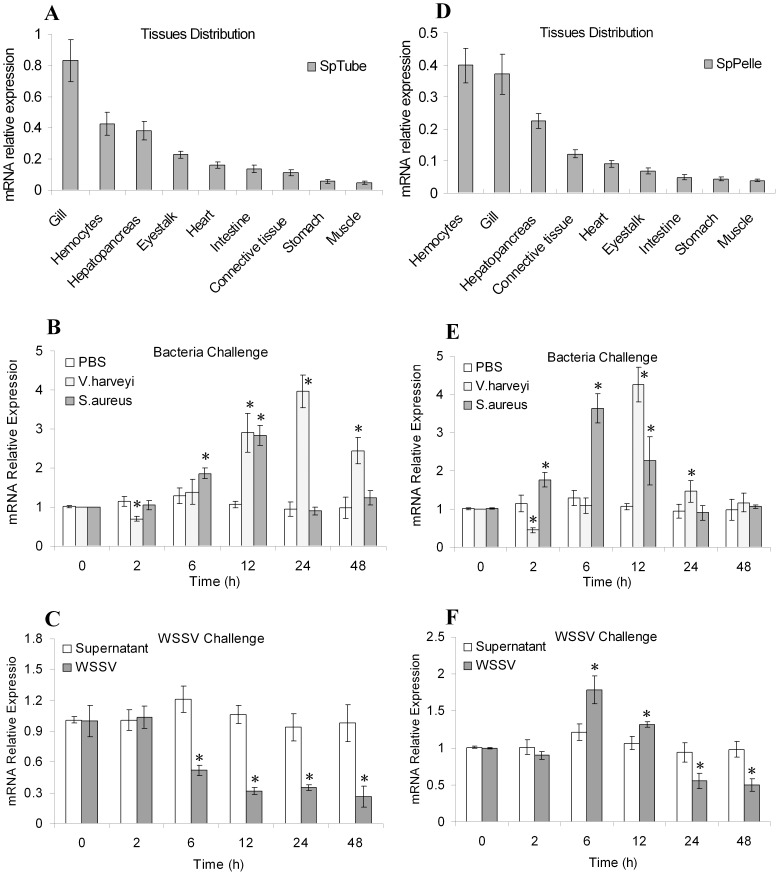
Tissue distributions and expression profiles after challenge with bacteria or WSSV. The transcripts of *SpTube* (A) and *SpPelle* (D) in different tissues were examined by qRT-PCR. The temporal expression level of *SpTube* in hemocytes after challenge with *V. harveyi* and *S. aureus* (B); and the expression patterns of *SpPelle* after challenge with *V. harveyi* and *S. aureus* (E). The temporal expression level of *SpTube* (C) and *SpPelle* (F) in hemocytes after challenge with WSSV. The asterisks indicate significant differences from the control (*: P<0.05).

qRT-PCR was performed to analyze the tissue distributions of *SpTube* and *SpPelle*. As illustrated in Fig. 8A, *SpTube* was mainly distributed in the gills, hemocytes, hepatopancreas, and eye stalks. *SpPelle* was highly expressed in hemocytes, gills, hepatopancreas, and connective tissues (Fig. 8D). In addition, the transcripts of these two genes could also be detected in all other examined tissues.

Given that both *SpTube* and *SpPelle* were highly expressed in hemocytes, we further examined the temporal expression post-injection with pathogens. After the challenge with *V. harveyi*, the *SpTube* transcripts in the hemocytes were up-regulated from 12 h to 48 h post-injection and elevated by approximately threefold at 24 h (Fig. 8B). Similarly, the *SpPelle* transcripts in the hemocytes were up-regulated at 12 h. They reached their highest levels (approximately threefold increase) at 24 h post-injection and then recovered to their normal levels at 48 h post-injection (Fig. 8E). Moreover, slight decreases were also found for both *SpTube* and *SpPelle* transcripts at 2 h post-injection. The expression of *SpTube* in the *S. aureus*-challenged experiments was remarkably elevated from 6 h to 12 h and then recovered gradually (Fig. 8B). The expression of *SpPelle* significantly increased from 2 h to 12 h after the challenge (Fig. 8E). These results imply that both *SpTube* and *SpPelle* may participate in the immunity against bacteria. Although *SpPelle* expression decreased from 24 h to 48 h after challenge with WSSV, it was elevated from 6 h to 12 h post-injection. By contrast, *SpTube* was down-regulated by WSSV injection from 6 h to 48 h, suggesting that *SpPelle* migh be also involved in antiviral immune responses (Figs. 8C and 8F).

### Recombinant Expression and Purification

**Figure 9:**
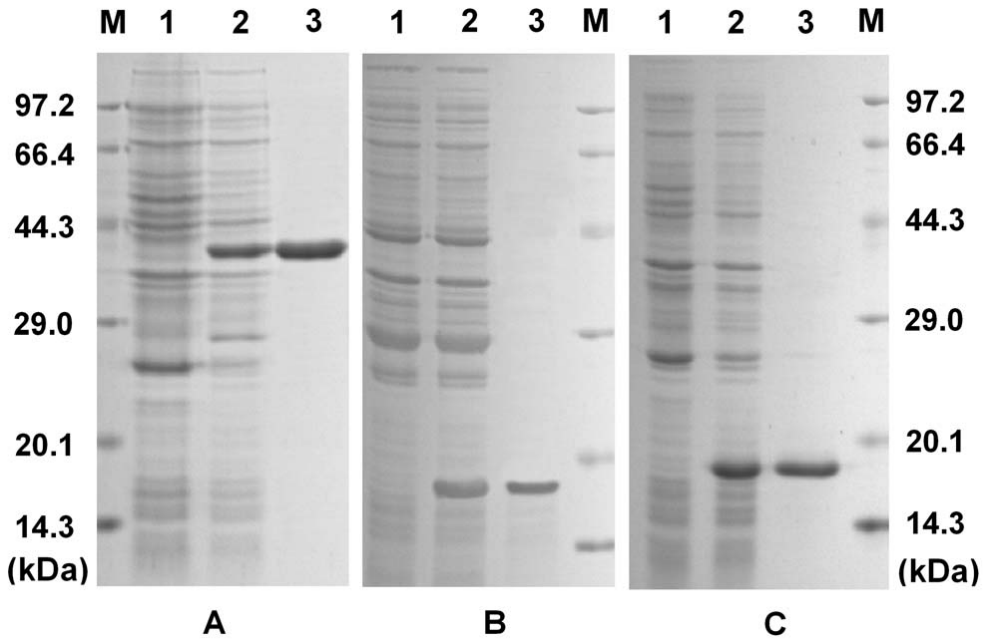
(A) SDS-PAGE analysis of recombinant *Sp*Tube-DD expressed in *Escherichia coli*. Lane M, protein marker; Lane 1, total protein obtained from *E. coli* expressing pGEX4T1-*Sp*Tube-DD; Lane 2, total protein obtained from *E. coli* expressing GST*-Sp*Tube-DD after IPTG induction; Lane 3, recombinant GST*-Sp*Tube-DD purified by glutathione Sepharose 4B chromatography. (B) SDS-PAGE analysis of recombinant *Sp*MyD88-DD expressed in *E*. *coli*. Lane 1, total protein obtained from *E. coli* expressing pET30a-*Sp*MyD88-DD; Lane 2, total protein obtained from *E. coli* expressing *Sp*MyD88-DD after IPTG induction; Lane 3, recombinant *Sp*MyD88-DD purified by His-tag resin chromatography. (C) SDS-PAGE analysis of recombinant *Sp*Pelle-DD expressed in *E*. *coli*. Lane 1, total protein obtained from *E. coli* expressing pET30a-*Sp*Pelle-DD; Lane 2, total protein obtained from *E. coli* expressing *Sp*Pelle-DD after IPTG induction; Lane 3, recombinant *Sp*Pelle-DD purified by His-tag resin chromatography.

*Sp*TubeDD was expressed as a soluble protein after IPTG induction. It was conveniently purified by glutathione Sepharose 4B chromatography. The purified protein comprised a DD (theoretical Mw, 13.2 kDa) and an approximately 26 kDa GST tag expressed by the plasmid pGEX4T1, which was roughly consistent with the size (approximately 39.2 kDa) of the unique band in the purified protein lane (Fig. 9A). Both *Sp*MyD88DD and *Sp*PelleDD were purified by His-Bind resin chromatography because each of these two recombinant proteins contained an additional *N*-terminal His Tag. Therefore, the theoretical Mw values of the purified *Sp*MyD88DD and *Sp*PelleDD were larger than those of their separate DDs, i.e., approximately 15.3 and 16.5 kDa, respectively, which were approximately consistent with their individual SDS-PAGE results (Figs. 9B and 9C).

### Binding Activity of SpTube DD

**Figure 10:**
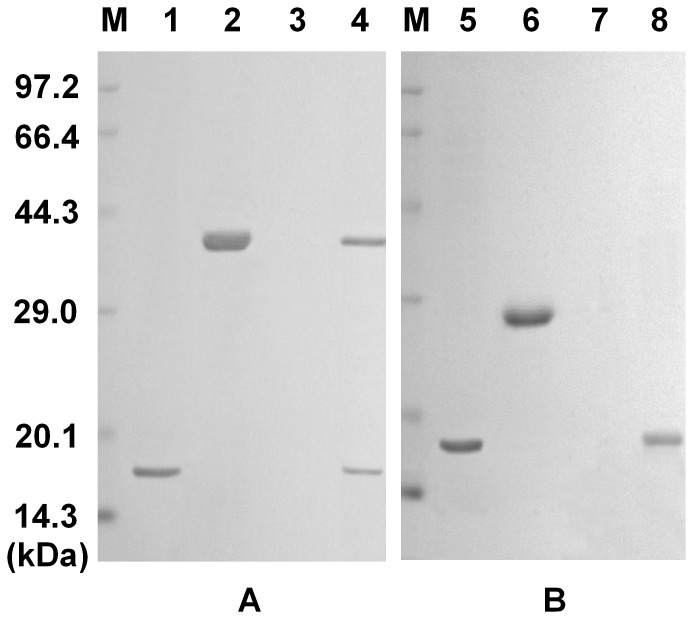
Pull-down assays to test the binding activity of *Sp*Tube death domain (TubeDD) with *Sp*MyD88 death domain (MyDD). (A) Recombinant MyDD was bound to His Bind resin, to which purified GST-TubeDD protein was added. MyDD and TubeDD were eluted simultaneously with elution buffer but not with wash buffer. (B) Recombinant MyDD was added to His Bind resin, to which GST alone was added as a control. Only MyDD was eluted by elution buffer. M, protein markers; Lane 1, recombinant MyDD; Lane 2, GST-TubeDD; Lane 3, collection of wash buffer; Lane 4, collection of elution buffer; Lane 5, recombinant MyDD; Lane 6, GST; Lane 7, collection of wash buffer; Lane 8, collection of elution buffer.

**Figure 11:**
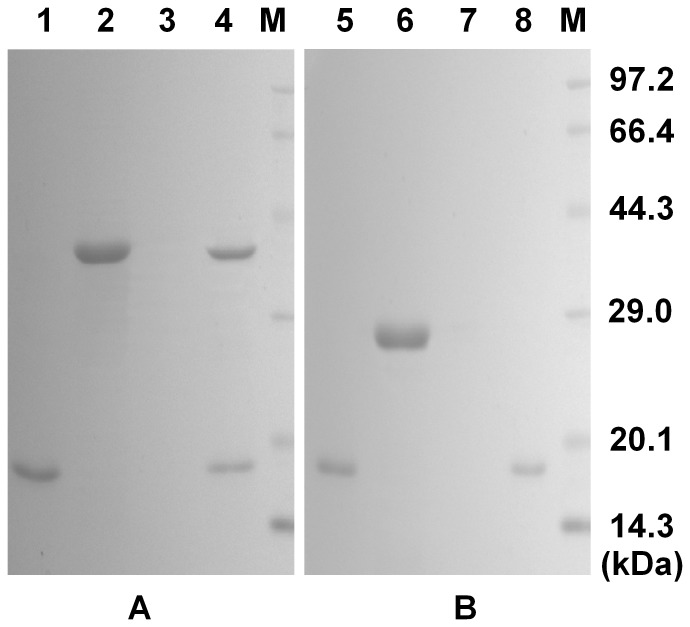
Pull-down assays to test the binding activity of *Sp*Tube death domain (TubeDD) with *Sp*Pelle death domain (PelleDD). (A) Recombinant PelleDD was bound to His Bind resin, to which purified GST-TubeDD protein was added. PelleDD and TubeDD were eluted simultaneously with elution buffer but not with wash buffer. (B) Recombinant PelleDD was added to His Bind resin, to which GST alone was added as a control. Only PelleDD was eluted by elution buffer. M, protein markers; Lane 1, recombinant PelleDD; Lane 2, GST-TubeDD; Lane 3, collection of wash buffer; Lane 4, collection of elution buffer; Lane 5, recombinant PelleDD; Lane 6, GST; Lane 7, collection of wash buffer; Lane 8, collection of elution buffer.

The potential binding activities of *Sp*TubeDD with *Sp*MyD88DD and *Sp*PelleDD were examined by a pull-down assay. GST-tagged *Sp*TubeDD but not GST protein could bind to his-tagged *Sp*MyD88DD (Fig. 10). In addition, His-tagged *Sp*PelleDD but not GST protein could interact with GST-tagged *Sp*Tube DD (Fig. 11). These results indicate that *Sp*Tube DD could interact with both *Sp*MyD88DD and *Sp*PelleDD.

## Discussion

In the past few years, although increasing evidence provided by different research groups suggests that TLR signaling pathways may exist in crustaceans, no such pathway has been established in any crustacean species to date [33]. One of the original causes is the fact that some essential components of TLR signaling pathways, such as Tube and Pelle homologs, have not been identified or well characterized in crustaceans. In this study, we identified the first pair of crustacean homologs of Tube and Pelle, namely, *Sp*Tube and *Sp*Pelle, and analyzed their individual characteristics and likely immune functions. *Sp*Tube was found to interact with both *Sp*MyD88 and *Sp*Pelle, which are involved in the immunity against both Gram-negative and Gram-positive bacteria.

In *Drosophila*, Tube and Pelle are two closely related but distinct components of Toll signaling pathway. A former study revealed that *Tube* and *Pelle* may arise by duplication and divergence of an ancestral gene [32]. One remarkable difference between these two protein molecules is that Tube DD is bivalent, which mediates specific and essential interactions with dMyD88 at one binding surface and with Pelle at the other [32]. In our study, we found that it was *Sp*Tube DD, instead of *Sp*Pelle DD, that shared several common conserved core residues at two separate binding surfaces with Tube DD. Furthermore, these conserved residues showed similar spatial locations with those in Tube. These results indicate that *Sp*Tube, similar to *Drosophila* Tube, may also be a bivalent protein that possesses potential binding activity with both its upstream component *Sp*Myd88 and downstream component *Sp*Pelle.

In addition, although *Drosophila* Tube lacks the kinase domain, most of the Tube and Pelle homologs from insects and humans are a pair of kinase proteins with a kinase domain in their individual C-terminals: one is an RD kinase and the other is a non-RD kinase. A previous study demonstrated that insect Tube-like kinases and human IRAK4 are RD kinases, whereas Pelle, insect Pelle-like kinases, and human IRAK1 are non-RD kinases [32]. In humans, the presence of a pair of protein kinases is involved in activating the downstream components through both phosphoregulation and phosphorelay activities [12]. Given that *Sp*Tube is an RD kinase and *Sp*Pelle is a non-RD kinase, *Sp*Tube and *Sp*Pelle may be their individual counterparts of human IRAK4 and IRAK1 in mud crab. Thus, the mechanism by which these two proteins function in the likely-existing TLR signaling pathway may be similar to that of their individual counterparts in humans.

Until recently, *Drosophila* Tube and Pelle have been considered as an orthologous pair to human IRAK-4 and IRAK-1 [32]. However, before this report, no mammalian counterpart for *Drosophila* Tube has been identified because of the lack of a catalytic domain for Tube, which once had been a long-standing confusion regarding Toll signaling in flies and mammals. Furthermore, IRAK4 was once considered as the counterpart of Pelle [34]. This assumption may mislead the following study about how to identify these two genes in other species. For example, the recently identified crustacean *Lv*Pelle [35], which shares higher identity with human IRAK4 (29.3%) and *Drosophila* Pelle (27.0%) than with *Drosophila* Tube (14.7%) and contains a DD and a kinase domain, has been considered as a “Pelle” homolog. However, this protein should be a Tube rather than a Pelle homolog because its DD possesses the typical characteristics of a Tube homolog, i.e., a bivalent DD and a RD kinase domain. Furthermore, *Lv*Pelle has shown close evolutionary relationship with Tube homologs, such as human IRAK4 and *Sp*Tube.

In addition to the DD and kinase domain, many insect Tube-like proteins contain several copies of the conserved sequence motif “Tube repeat motif,” which is also a distinctive feature for Tube molecules because this motif exists only in insect Tubes but not in Pelle or Pelle-like proteins in previous reports [32]. Intriguingly, two copies of the conserved repeat motifs were found in *Sp*Pelle but not in *Sp*Tube. This finding indicates that the conserved repeat motifs were no longer a unique feature for Tube and Tube-like molecules, which were once considered as a marker to distinguish Tube from Pelle. Although we did not test the exact function of these conserved repeat motifs, a previous report showed that these repeat motifs of *Drosophila* Tube participate in the binding activity to Dorsal and that Pelle can bind to Dorsal [36]. Based on this result, we speculated that *Sp*Pelle binds to SpDorsal and that *Sp*Pelle repeat motifs contribute to this binding activity. This speculation further implies that *Sp*Tube and *Sp*Pelle have important functions in the signaling transduction of the possibly-existing TLR signaling pathway in mud crab.

*LvPelle* (now identified as *Tube* ortholog) in hepatopancreas is up-regulated by *Vibrio alginolyticus* [35]. In the present study, *SpTube* and *SpPelle* were elevated by *V. harveyi*, another species of Gram-negative bacteria. These two molecules can also be induced by the Gram-positive bacteria *S. aureus*. These results indicate that both *Tube* and *Pelle* orthologs of crustaceans may participate in the immunity against both Gram-negative and Gram-positive bacteria. In addition, *Sp*Tube DD and *Sp*Pelle DD have similar 3D structures with their respective counterparts of *Drosophila* Tube and Pelle. Furthermore, *Sp*Tube was also proven capable of binding to *Sp*MyD88 and *Sp*Pelle via its bivalent DD. These findings suggest that *Sp*MyD88, *Sp*Tube, and *Sp*Pelle may form a trimeric complex involved in the immunity against both Gram-negative and Gram-positive bacteria.

In the *Drosophila* Toll signaling pathway, dMyD88 recruits its upstream-activated Toll receptor and downstream cytosolic adaptor Tube [8], thereby providing a platform to permit Pelle incorporation and forming a trimeric complex (dMyD88-Tube-Pelle). The forming of this complex further activates Pelle, which consequently activates Dorsal [37]. Similarly, in our previous work, we proposed a plausible antibacterial model mediated by *Sp*Toll and *Sp*MyD88. In this model, *Sp*Toll cooperating with *Sp*MyD88 is involved in the immunity against Gram-negative bacteria [25]. In this study, we further complement this likely antibacterial model. Upon challenge with Gram-negative bacteria, *Sp*MyD88, which pre-anchored the plasma membrane via its CTE domain, would recruit its upstream-activated *Sp*Toll and downstream cytosolic adaptor *Sp*Tube. As a scaffold protein, *Sp*Tube would further recruit *Sp*Pelle, thereby forming a trimeric complex. In this case, *Sp*Pelle may be finally activated via the kinase domain of *Sp*Tube in a similar manner to IRAK1 activated by IRAK4 [13].

In conclusion, we identified the first pair of Tube and Pelle homologs in crustaceans and further analyzed their individual characteristics. *Sp*Tube is a RD kinase with a bivalent DD and a C-terminal kinase domain, whereas *Sp*Pelle is a non-RD kinase with an N-terminal DD, two repeat motifs, and a C-terminal kinase domain. *Sp*Tube was quite different from *Drosophila* Tube in sequence structure, which contains a DD and repeat motifs but lacks a C-terminal kinase domain. The conserved “Tube repeat motif” was once found in only insect Tube or Tube-like molecules; however, the similar “Tube repeat motif” appeared in *Sp*Pelle. These findings well characterize *Sp*Tube and *Sp*Pelle, as well as the Tube and Pelle homologs in crustaceans and insects. In addition, *Sp*Tube can bind to both *Sp*MyD88 and *Sp*Pelle, which are involved in the immunity against both Gram-negative and Gram-positive bacteria. Therefore, these findings further complement our previously proposed antibacterial model that may exist in mud crab.
